# Properties of an Oral Preparation Containing a Chitosan Salt

**DOI:** 10.3390/molecules14020755

**Published:** 2009-02-13

**Authors:** Yoshifumi Murata, Youko Kodama, Daijirou Hirai, Kyouko Kofuji, Susumu Kawashima

**Affiliations:** Faculty of Pharmaceutical Science, Hokuriku University, Ho-3, Kanagawa-machi, Kanazawa 920-1181, Japan; E-mails: 2007m006@stu.hokuriku-u.ac.jp (Y.K.), k-kofuji@hokuriku-u.ac.jp (K.K.)

**Keywords:** Chitosan, 2-(4-Chlorophenoxy)-2-methylpropionic acid, Bile acid adsorption, Drug release, Hyperlipidemia.

## Abstract

The 2-(4-chlorophenoxy)-2-methylpropionic acid (CMP) salt of chitosan (CS), CS-CMP, and that of a CS derivative (CP), were prepared and their ability to adsorb bile acids investigated. CS-CMP and CP-CMP rapidly adsorbed taurocholate (TCA) and glycocholate (GCA) when these bile acids were present together in the medium, with simultaneous release of CMP. A secondary bile acid, taurodeoxycholate, was preferentially adsorbed over TCA and GCA. Alginate gel beads containing CS-CMP did not differ from CS-CMP alone in their manner of bile acids take up. Furthermore, oral administration of CS-CMP to rats resulted in decreased serum cholesterol and triacylglycerol levels for two weeks. Therefore, CS-CMP, as well as a vehicle containing CS-CMP, might be a useful agent with which to treat hyperlipidemia.

## Introduction

Chitosan (CS) is an attractive agent for drug development given its function in the gastrointestinal tract and its intrinsic safety when taken orally. CS is a cationic polymer, and a number of CS derivatives have been developed and used as anion-exchange resins [1,2]. Recently, CS has been examined as an alternative therapy, since oral administration of CS leads to decreased serum cholesterol levels [3,4,5]. One mechanism by which CS might decrease cholesterol levels is by adsorption of bile acids (BAs) [6]. BAs are secreted in the gastrointestinal tract, primarily as glycine or taurine conjugates (primary BAs), after which secondary BAs, such as deoxycholate, are produced from primary BAs by intestinal microorganisms. BAs are re-cycled via the enterohepatic circulation. Therefore, oral administration of an anion-exchange resin, such as cholestyramine, inhibits the enterohepatic circulation of BAs, thereby decreasing serum cholesterol levels [7]. In addition, a number of drugs capable of influencing lipid metabolism have been used in the treatment of hyperlipidemia [8,9,10]. For example, clofibrate, an ethyl ester of 2-(4-chlorophenoxy)-2-methyl- propionic acid (CMP) changed into CMP in the intestine and CMP is absorbed from the intestinal tract.

We have previously reported that a weak acid salt of CS adsorbs bile acids through electrostatic interactions [11,12]. Even though CMP is a weak acid with limited water solubility (0.85 mg/mL at 37 °C), it might be able to induce the formation of a CS salt. In the present study, we prepared an electrostatic complex between CS and CMP (a CMP salt of CS), called CS-CMP. A CS derivative, chitopal (CP), is utilized as a carrier for enzyme immobilization. We also produced a CMP salt of a CP derivative, CP-CMP, and investigated the adsorption of BAs secreted into the human intestine by CS-CMP or CP-CMP *in vitro*. In addition, CS-CMP was introduced into rat feed in order to investigate its effect on serum cholesterol and triacylglycerol levels. We also devised a pharmaceutical form of CS-CMP which can be swallowed, since CS is a fine powder and difficult to swallow. Therefore, CS-CMP was incorporated into calcium-induced alginate gel beads (Alg-Ca), and the potential of these beads to reduce hyperlipidemia following oral administration was examined *in vitro* [13].

## Results and Discussion

**Figure 1:**
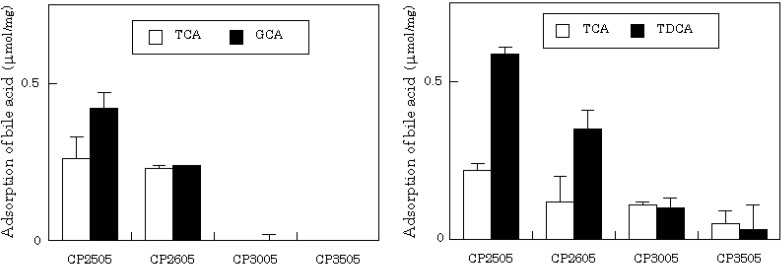
Adsorption of BAs by CP-CMP.

When sodium taurocholate, TCA and glycocholate, GCA (2 mM each) were both present in the medium, they were rapidly adsorbed by CS-CMP. For example, 0.19 ± 0.01 µmol/mg and 0.14 ± 0.04 µmol/mg of TCA and GCA were adsorbed after 30 min, respectively. At the same time, CMP (0.24 ± 0.00 µmol/mg) was released from the powder. The combined mixture of CS and CMP did not adsorb BAs. Four CP-CMP compounds prepared with CP2505, CP2605, CP3005 and CP3505 were also tested. The extent of BA adsorption was affected by the number and/or structure of amino groups within each of the compounds. The capacity for adsorption declined as fewer amino groups were present, as shown in Figure 1. Apart from CP2505, which contained a quaternary ammonium salt, limited adsorption of BAs was observed with the CP compounds alone, in the absence of CMP. These results show that CS-CMP may release CMP by ion-exchange within the gastrointestinal tract when the CS salt is administered orally. CP-CMP also adsorbed a secondary BA, taurodeoxycholate (TDCA). In particular, CP-2505 and CP-2605 preferentially adsorbed TDCA when TCA (2 mM) and TDCA (2 mM) were both present in the medium. Almost twice as much TDCA than TCA was adsorbed.

**Figure 2:**
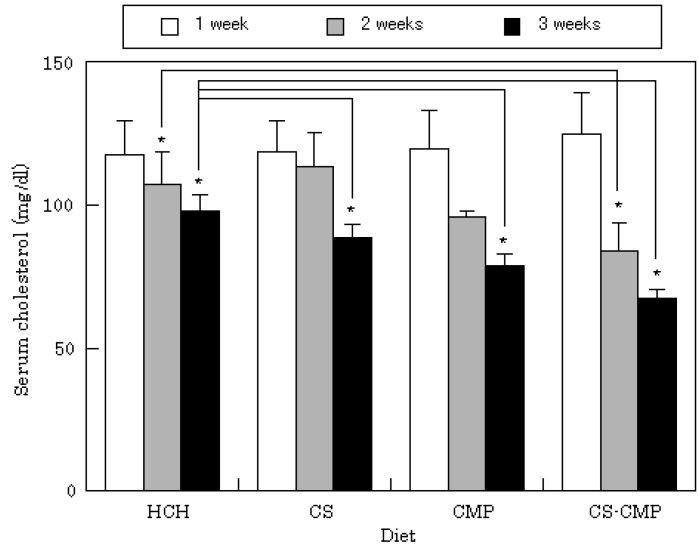
Changes in total serum cholesterol.

**Figure 3:**
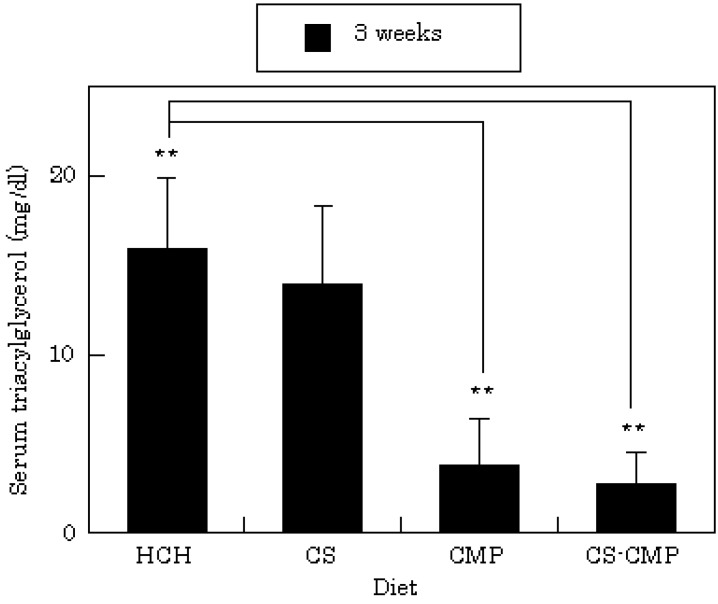
Effects of diet on serum triacylglycerol levels.

The food intake of rats was not affected by adding cholesterol or other components to the commercial powder diet (CRF-1). No changes in food intake were observed when CS, CS-CMP, or CMP were added to the HCH-diet, and no differences in effects on body weight were observed with the various diets. Among rats fed the HCH-diet, serum cholesterol levels were 97.9 ± 5.5 mg/dL (n=3) after 3 weeks. Serum cholesterol levels changed when chitosan-related compounds were added to the HCH-diet, as shown in Figure 2. In rats fed the CMP diet, mean cholesterol was 78.8 ± 4.2 mg/dL at 3 weeks (n=3). After 3 weeks, a decrease in serum cholesterol was also observed in the CS-diet group. A pronounced decrease was observed in rats in the CS-CMP group, with cholesterol levels of 83.8 ± 9.9 mg/dL at 2 weeks and 67.5 ± 3.2 mg/dL at 3 weeks. Oral administration of CS-CMP to rats also resulted in decreased serum triacylglycerol levels. As shown in Figure 3, triacylglycerol levels were 2.8 ± 1.7 mg/dL at 3 weeks in rats fed the CS-CMP diet, and almost five times this amount in rats fed the HCH-diet. This result may be due to the pharmacological activity of CMP released from CS-CMP in the gastrointestinal tract. Actually, the same effect was observed at 3 weeks in rats fed the CMP diet (triacylglycerol level: 3.8 ± 2.6 mg/dL). 

Results are expressed as mean values and the bars indicate S.D. (n=3).

Results are expressed as mean values and the bars indicate S.D. of three experiments. Significant differences between the two groups, * *p*<0.05.

Results are expressed as mean values and the bars indicate S.D. of three experiments. Significant differences between the two groups, ** *p*<0.05.

**Figure 4:**
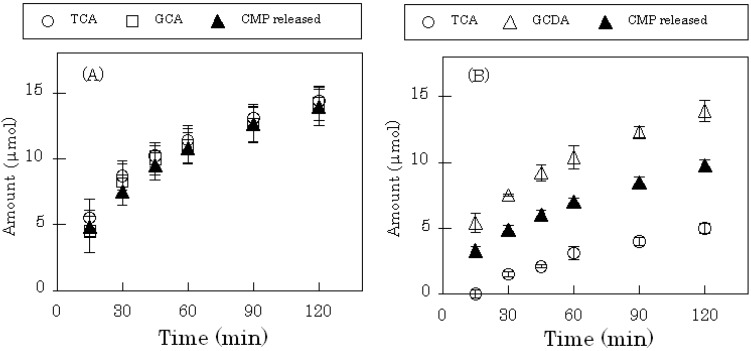
Uptake of BAs into dried Alg-Ca containing CS-CMP.

The Alg-Ca prepared in this study theoretically contains about 100 mg of CS, which was converted to the CMP salt within the gel matrix by autoclaving in CMP suspension. Alg-Ca gradually took up two types of BAs from solution, as shown in Figure 4. And the amount of CMP released from Alg-Ca gradually increased as more BAs were taken up. When TCA and GCA were both present in the medium, equal uptake by Alg-Ca was observed. However, preferential uptake of glycochenodeoxycholate, GCDA over TCA by Alg-Ca was observed when both were present in solution, with almost three times as much GCDA being taken up after 60 min.

(A) Initial BA concentration; 2 mM TCA, 2 mM GCA

(B) Initial BA concentration; 2 mM TCA, 2 mM GCDA

Results are expressed as means and the bars indicate S.D. (n=3).

## Conclusions

CS-CMP or Alg-Ca containing CS-CMP, as prepared in this study, were observed to demonstrate two useful properties within the gastrointestinal tract:(a) they released CMP, and (b) they adsorbed bile acids. In addition, oral administration of CS-CMP to rats decreased serum cholesterol and triglyceride levels. CS-CMP or Alg-Ca containing CS-CMP is potential agent for the treatment of hyperlipidemia, and the latter prepared in this study is a prototype for oral dosage form. Therefore, we shall make further improvements for the application to human being.

## Experimental

### General

**Figure 5:**
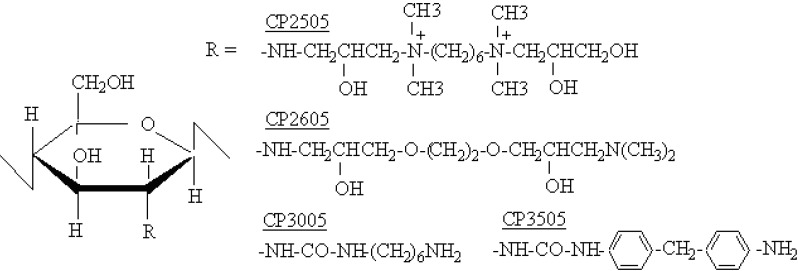
CP structures.

One type of CS (fine powder, molecular weight: 213,000 Da, degree of deacetylation: 75-85%) was obtained from Kimitsu Chemical Industries (Tokyo, Japan), and four types of CP (chitopearl-BCW) were obtained from Fujibouseki Ltd. Co. (Tokyo, Japan). The structures of these are shown in Figure 5. The diameters of the CP compounds ranged from 0.1-1.0 mm. CMP was purchased from Aldrich Chem. Co. (Milwaukee, WI, USA). TCA, GCA, TDCA, and GCDA were all purchased from Nacalai Tesque (Kyoto, Japan). All of the other chemicals were of reagent grade.

### Preparation of CS-CMP

CS-CMP was prepared as follows: CS (10 g) and CMP (1 g) were added to water (250 mL) and stirred for 1 day at room temperature. The suspension was then filtrated with a glass filter (17G), washed twice with ethanol (100 mL) to remove any CMP remaining in the residue. The powder obtained was dried for 8 h on a dish, followed by desiccation in a vacuum in the presence of P_2_O_5_. 

### Preparation of CP-CMP

Each CP compound was purchased as an ion-exchange resin (diameter; 0.5 mm). It was washed with distilled demineralized water and ethanol using the centrifuge and then it was dried at room temperature. Dried CP compounds were treated with the suspension of CMP as follows: CP (0.3 g) and CMP (0.03 g) were added to water (50 mL) and stirred for 1 day at room temperature. The suspension was then centrifuged at 3,000 rpm for 5 min. The precipitate was collected and washed twice with distilled demineralized water and ethanol (50 mL), dried on a dish, and desiccated under a vacuum in the presence of P_2_O_5_. 

### Preparation of Alg-Ca containing CS-CMP

Alg-Ca containing CS-CMP, Alg-CS, was prepared as follows: CS (0.5 g) was dispersed in 1(w/w)% sodium alginate solution (9.5 g) with agitation. Two grams of the suspension containing 5(w/w)% CS was dropped into 0.1 M CaCl_2_ (10 mL) using a glass pipette, and left to stand at room temperature for 1 h. Next, the spherical hydrogel beads (diameter; 4 mm) were transferred to distilled demineralized water (50 mL) containing CMP (0.05 g), which was autoclaved at 121 °C for 15 min. The Alg-Ca (hydrogel bead) was then taken out and dried at 35 °C for 8 h on a dish, followed by vacuum treatment in a desiccator in the presence of P_2_O_5_. 

### Test of CMP Release and bile acid adsorption

Fifteen milliliters of a 2 mM bile acid solution were placed into L-shaped glass tubes and maintained at 37 °C. Ten milligrams of CS-CMP or dried Alg-CS corresponding to 2 g of hydrogel, were added to the tubes and shaken 67 times per min. A 0.2 mL aliquot of each solution was removed periodically to determine the amount of CMP and bile acid by HPLC analysis. The HPLC system had an LC-6A pump (Shimadzu, Kyoto, Japan), a packed column (Mightysil RP-18 GP, 150 mm x 4.6 mm, Kanto Chem. Co., Tokyo, Japan), and a SPD-6A UV detector (Shimadzu, Japan). HPLC was conducted at ambient temperature, using an eluent containing methanol, 30 mM phosphate buffer (pH 3.4), and acetonitrile (6:3:1), at a flow rate of 0.6 mL/min. The detector wavelength was set at 230 nm. The amount of bile acid taken up into the Alg-CS was calculated based on the amount of bile acid added and that remaining at sampling time. All tests were performed in triplicate.

### Animal study

The powdered feed given to the rats was the certified diet (CRF-1) and was obtained from Oriental Yeast Co. (Japan). The composition of the diet, including its cholesterol content (HCH-diet), is shown in Table 1. HCH-diet containing CS (CS-diet) or CS-CMP (CS-CMP-diet) was prepared by adding 4% CS or 4% CS-CMP to the HCH-diet. Each diet was mixed well and given to rats after sifting through a sieve (710 µm). 

**Table 1:** Composition of HCH-Diet.

Component	(%)
CRF-1	96.8
Cholesterol	1.0
Olive oil	2.0
Sodium cholate	0.2

The experimental protocol was approved by the Ethics Committee at Hokuriku University. Male Wistar rats (5 weeks) were housed individually in stainless-steel wire-bottomed cages in an air-conditioned room, and were allowed free access to food and water for 3 weeks. The food intake and body weight of each rat was measured daily. All rats were fed the HCH-diet for 1 week, followed by each of the various test diets. Blood samples from the rats were collected from the superficial lateral caudal vein after 1, 2 and 3 weeks. Blood was clotted at room temperature, after which it was centrifuged in an ultracentrifuge (Kokusan H-1300, Japan) at 3000 rpm for 10 min. Serum was separated and total cholesterol and triacylglycerol levels were measured using an enzymatic method and a commercial kit, respectively. If necessary, data were compared using Student’s two tailed *t*-test, and differences were considered significant when *P* < 0.05.
